# Development and Evaluation of an Augmented Artificial Intelligence Model for Urogynecology Queries

**DOI:** 10.1007/s00192-025-06446-x

**Published:** 2025-12-09

**Authors:** Madeline K. Moureau, Berkley Davis, Christopher X. Hong

**Affiliations:** 1 University of Michigan Medical School, 1540 E Medical Center Dr, Ann Arbor, MI 48109 USA; 2 Department of Obstetrics and Gynecology, University of Michigan, Ann Arbor, MI USA

**Keywords:** Artificial intelligence, Patient education, Pelvic floor disorders, Retrieval-augmented, Urogynecology

## Abstract

**Introduction and Hypothesis:**

The objective was to develop a retrieval-augmented ChatGPT model grounded in evidence-based patient education materials and compare its performance against the standard ChatGPT model in responding to common urogynecology patient questions in this pilot study.

**Methods:**

We developed a retrieval-augmented ChatGPT-4.0 model that prioritized content from International Urogynecological Association patient information leaflets. Ten commonly asked patient questions were submitted to both the standard and retrieval-augmented models. Six board-certified urogynecologists evaluated responses using the validated Quality Analysis of Medical Artificial Intelligence (QAMAI) tool, which assesses accuracy, clarity, relevance, completeness, usefulness, and sources. Total and domain-specific QAMAI scores were compared using the Wilcoxon signed-rank test, and a sensitivity analysis was performed, excluding the unblinded Source domain.

**Results:**

The retrieval-augmented model demonstrated significantly higher total QAMAI scores than the standard model (median 22 [interquartile range, IQR, 19–25] vs 16 [IQR 13–18], *p* < 0.01) and outperformed the standard model in all six domains. In the sensitivity analysis, the retrieval-augmented model maintained significantly higher performance (18 [IQR 16–20] vs 14.5 [IQR 11–17], *p* < 0.01). Clinician raters preferred the retrieval-augmented model in 81% of responses.

**Conclusions:**

Grounding AI tools in vetted patient education materials significantly improved the quality of ChatGPT-generated responses in urogynecology. Retrieval-augmented models offer a promising approach to enhance patient education and promote patient-centered care.

**Supplementary Information:**

The online version contains supplementary material available at 10.1007/s00192-025-06446-x

## Introduction

Patients increasingly turn to the internet to access health information, using a variety of digital tools to better understand their symptoms, diagnoses, and treatment options, including pelvic floor disorders [1–3]. These tools include general search engines, health-specific websites, social-media platforms, and online discussion forums [4]. More recently, artificial intelligence (AI) large language models, such as ChatGPT, have emerged as new sources of medical information, offering real-time, conversational responses to user-inputted questions [5]. Despite their accessibility and appeal, these models may disseminate inaccurate or incomplete information, raising concerns about their reliability as tools for patient education [6, 7].

To support informed decision-making, health care providers typically provide counseling during clinic visits and may supplement this with written materials, such as patient-education leaflets. In urogynecology, professional societies have developed evidence-based patient education leaflets to promote accurate and accessible information about pelvic floor disorders [8, 9]. As AI tools such as ChatGPT gain traction for answering health-related queries, there exists a largely untapped opportunity to leverage these high-quality, vetted resources to guide and improve AI-generated responses. Despite the increasing use of AI in patient education, limited research has explored how traditional educational materials can be integrated into AI frameworks to improve the reliability of AI-generated medical content [10, 11].

To address this gap, we developed a retrieval-augmented ChatGPT model that integrates vetted patient-education materials. This model was designed to prioritize expert-developed content while generating responses to patient questions. The objective of this pilot study was twofold: to develop the retrieval-augmented ChatGPT model, and to compare its performance against the standard ChatGPT model (control) in responding to common patient questions in urogynecology.

## Materials and Methods

This study followed the Chatbot Health Advice Reporting Template guidelines for evaluating generative AI systems that provide health information. The study was reviewed and deemed not regulated by our Institutional Review Board (HUM00265752) because all data analyzed were publicly available educational materials, and subspecialist raters served solely as expert evaluators.

A customized, retrieval-augmented ChatGPT-4.0 (gpt-4-turbo-2024–11-01) model was developed in November 2024 using publicly available patient information leaflets from the International Urogynecological Association (IUGA). Both models were based on the proprietary ChatGPT-4.0 large language model developed by OpenAI and used the default system parameters.

The retrieval-augmented model was created using ChatGPT’s *MyGPT* interface with integrated document retrieval. All publicly available IUGA patient information leaflets were uploaded as attachments to the knowledge base of the model, which enabled semantic retrieval of relevant content during response generation. When a user query was entered, the model performed an internal embedding-based similarity search to identify the most relevant IUGA leaflets. The system automatically searched and referenced relevant sections of the corpus when responding to user questions. Model instructions established guardrails to prevent citation of non-IUGA sources and ensure alignment with the uploaded educational content. Hyperlinks to IUGA materials were included where appropriate to facilitate further reading.

In January 2025, to evaluate the performance of the model, ten questions commonly asked by urogynecology patients were selected through consensus among urogynecologists. The question domains were derived directly from the IUGA patient information leaflets that formed the knowledge base of the retrieval-augmented model. These fact sheets comprehensively cover the major topics relevant to patient counseling in urogynecology, including overactive bladder management, prolapse treatment options, risks of surgical procedures (e.g., mid-urethral slings, sacral neuromodulation), use of vaginal estrogen, pessary care, urodynamic testing, and prevention of recurrent urinary tract infections. Each question was reviewed and refined by a board-certified urogynecology subspecialist to ensure clarity, clinical accuracy, and real-world relevance based on clinical practice. A full list of prompts and corresponding model responses is provided in the Supplementary File.

**Fig. 1:**
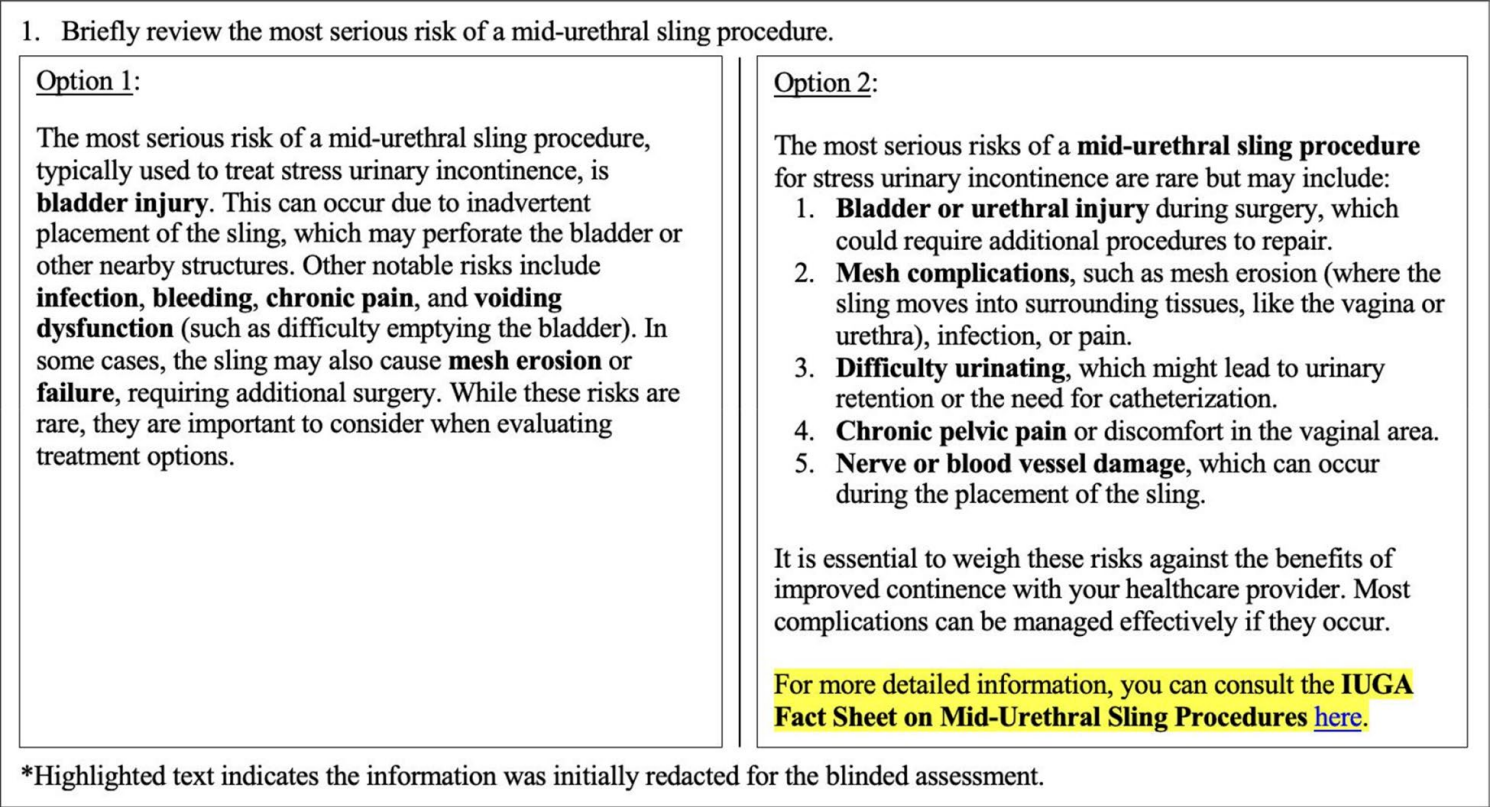
Example question with generated responses from both standard and retrieval-augmented artificial intelligence models

Model responses were collected and reviewed under blinded conditions, as highlighted in Fig. 1. All source-identifying elements, including hyperlinks and model identifiers, were removed to ensure that reviewers were blinded when assessing the first five Quality Analysis of Medical Artificial Intelligence (QAMAI) domains (accuracy, clarity, relevance, completeness, and usefulness; Table 1) as well as when indicating which response they preferred. As the Sources domain inherently reveals model identity, raters were subsequently unblinded and asked to evaluate the quality of source provision and referencing separately. Six board-certified urogynecology subspecialists, with 4 to over 40 years in practice, rated each response using the QAMAI tool, a validated instrument that uses a five-point Likert scale to assess the quality of health information, with higher scores indicating greater overall reliability [12]. The QAMAI tool includes six domains: accuracy, clarity, relevance, completeness, usefulness, and sources. Total and domain-specific QAMAI scores were calculated by summing individual item scores. Overall scores were categorized as follows: 6–11 (poor quality), 12–17 (fair quality), 18–23 (good quality), 24–29 (very good quality), and 30 (excellent quality) [12]. All responses were additionally screened for potentially harmful, biased, or misleading content, and none was identified.

**Table 1:** The Quality Analysis of Medical Artificial Intelligence (QAMAI) tool scoring system

Domain	Strongly disagree	Disagree	Neutral	Agree	Strongly agree
Accuracy: accurate and up-to-date information provided	1	2	3	4	5
Clarity: clear and understandable response	1	2	3	4	5
Relevance: information is relevant and answers question	1	2	3	4	5
Completeness: response answers the entire question	1	2	3	4	5
Sources: reliable sources and references provided	1	2	3	4	5
Usefulness: response meets the user’s health information	1	2	3	4	5

The sample size was determined pragmatically based on the number of urogynecology subspecialists available at our institution who were not involved in model development (*n* = 6). As a pilot study, this number was sufficient to enable a preliminary, controlled comparison across models and to generate reliability estimates to inform future larger-scale evaluations. Inter-rater reliability for QAMAI scoring among the six raters was assessed using a two-way random-effects intraclass correlation coefficient (ICC 2, *k*) to evaluate scoring consistency. ICC scores were interpreted as follows: < 0.20 (poor reliability), 0.20–0.40 (fair reliability), 0.41–0.60 (moderate reliability), 0.61–0.80 (good reliability), and > 0.80 (excellent reliability) [13].

Total and domain-specific QAMAI scores of the models were compared using the Wilcoxon signed-rank test. As the Sources domain could not be blinded, a sensitivity analysis was performed on the total QAMAI score, excluding this domain, to assess potential bias. Given the exploratory nature and limited sample size of this pilot study, formal multiplicity adjustments were not applied. Analyses were performed using RStudio, and a *p* value less than 0.05 was considered statistically significant.

## Results

The retrieval-augmented model demonstrated significantly higher overall performance, as measured by the validated QAMAI tool. The median total QAMAI score was 22 (interquartile range [IQR] 19–25) for the retrieval-augmented model, compared with 16 (IQR 13–18) for the standard model (*p* < 0.01; Table 2), representing a shift from “fair” to “good” quality based on established QAMAI score thresholds. The retrieval-augmented model outperformed the standard model across all six QAMAI domains, with the largest difference observed in the Sources domain (5 [IQR 4–5] vs 2 [IQR 1–2.5], *p* < 0.01).

**Table 2:** Quality of urogynecology patient information provided by the standard and retrieval-augmented artificial intelligence models, as assessed using the Quality Analysis of Medical Artificial Intelligence (QAMAI) tool

	Standard model	Retrieval-augmented model	*p* value*
QAMAI domain			
Accuracy	3 (2–4)	4 (3–4)	0.04
Clarity	3 (2–4)	4 (4–5)	< 0.01
Completeness	3 (2–4)	4 (3–4)	0.04
Sources	2 (1–2.5)	5 (4–5)	< 0.01
Relevance	4 (3–4)	4 (4–5)	< 0.01
Usefulness	3 (2–4)	4 (3–4)	< 0.01
QAMAI total	16 (13–18)	22 (19–25)	< 0.01
QAMAI total, without “References” domain score	14.5 (11–17)	18 (16–20)	< 0.01

Data are presented as median (interquartile range)

*Wilcoxon signed-rank test

In the sensitivity analysis, excluding the References domain, the retrieval-augmented model continued to demonstrate a significantly higher total QAMAI score than the standard model (18 [IQR 16–20] vs 14.5 [IQR 11–17], *p* < 0.01). Under blinded conditions, raters preferred the responses of the retrieval-augmented model in 81% of cases.

Inter-rater reliability for QAMAI scoring among the six urogynecology subspecialists was good to excellent, with ICC (2, 6) = 0.74 (95% CI 0.40–0.92) and ICC (3, 6) = 0.85 (95% CI 0.64–0.96).

## Discussion

In this pilot study, we developed and evaluated a retrieval-augmented ChatGPT model aimed at improving the quality of AI-generated patient education content in urogynecology. Responses from the retrieval-augmented model, grounded in patient information leaflets from a leading urogynecological association, were generally more structured, clinically precise, and aligned with guideline terminology, whereas the standard model produced broader, less detailed summaries that sometimes omitted key contextual or management information. Consistent with these qualitative differences, the retrieval-augmented model achieved significantly higher QAMAI scores, from 16 to 22, representing a meaningful transition from “fair” to “good” quality. These findings underscore the practical relevance of these improvements beyond statistical significance and importantly, remained consistent in sensitivity analyses that excluded the Sources domain. Urogynecologists preferred the retrieval-augmented model in 81% of cases, suggesting that grounding AI responses in expert-developed educational materials produces content that is more consistent with clinical consensus and better suited for patient counseling.

Large language models have become increasingly common in gynecology, with studies demonstrating their ability to generate high-quality responses in areas such as urogynecology, fibroid management, and cervical cancer screening [10, 14, 15]. More recently, medical specialties have begun to explore retrieval-augmented models as a way of enhancing AI-generated content. Kelly et al. developed a retrieval-augmented model for type 2 diabetes care grounded in patient education materials and found that it produced clinically accurate responses, although it was not directly compared with a standard model [16]. In a separate diabetes study, a retrieval-augmented model improved accuracy by approximately 12% compared with a standard version and also enhanced the comprehensiveness and understandability of its responses [17]. Similarly, in the field of neurology, a retrieval-augmented model called NeuroBot resulted in significant improvements in response accuracy, relevance, and completeness relative to a standard model [18]. These findings from other medical specialties align with our results and reinforce the potential of retrieval-augmented models to improve AI-generated health information. To our knowledge, this is the first study to evaluate a retrieval-augmented AI model in urogynecology.

Retrieval-augmented AI holds promise as a tool to supplement clinical conversations and improve health literacy in pelvic floor disorders. Although traditional patient education leaflets contain accurate and evidence-based information, they are static, non-interactive, and often written at a reading level that may not be accessible to all patients [19, 20]. Our findings demonstrate that retrieval-augmented models can bridge this gap by combining the adaptability and engagement of AI with expert-vetted materials. These models can tailor responses to individual questions, link to trustworthy sources, and potentially reinforce clinician explanations before or after visits. In clinical practice, such models could function as clinician-endorsed, post-visit educational tools, analogous to patient information leaflets but in an interactive format. By providing consistent, evidence-based information aligned with professional-society resources, these tools could improve patient comprehension and promote informed engagement in care. From a medico-legal perspective, these systems should be used as adjuncts to, not replacements for, clinician judgment and communication, with appropriate oversight to ensure accuracy and transparency.

Beyond improving informational quality, retrieval-augmented AI models may have meaningful implications for patient outcomes. By providing accurate, guideline-based information in an accessible conversational format, such models could help to counteract misinformation that patients frequently encounter online. Access to consistent, trustworthy explanations may also strengthen adherence to treatment plans by reinforcing clinician counseling. Furthermore, these interactive systems may empower patients to engage more actively in their care by promoting self-education, confidence, and shared decision-making. Compared with static leaflets, conversational models can adapt responses to individual concerns, improving comprehension and recall, factors shown to influence satisfaction, adherence, and long-term health-behavior change. Future research should evaluate how retrieval-augmented systems affect patient understanding, adherence, and health-related decision making in real-world clinical contexts. Although health literacy was not assessed in this study, future planned work will evaluate patient usability, comprehension, and trust when we are also able to measure and account for individual health literacy levels.

A strength of this study is its rigorous methodology, which included blinded, head-to-head comparisons between models using the QAMAI tool, a validated instrument specifically designed to assess the quality of health information. Unlike prior studies in gynecology that focused solely on evaluating existing large language models, we developed and tested a retrieval-augmented model designed to improve upon the standard ChatGPT model. Although the Wilcoxon signed-rank test was suitable for the study design, the relatively small sample size and limited number of patient-facing questions restrict statistical power and generalizability. Nevertheless, this targeted design enhanced the practical relevance of our findings by addressing real-world information needs, enabling controlled comparisons across models, and capturing a broad range of commonly encountered topics in urogynecology, including incontinence, prolapse, surgical procedures, and pessary care.

In addition, this study has several limitations. Because the Sources domain revealed model identity, some bias was possible; however, a sensitivity analysis excluding this domain yielded consistent findings, suggesting limited impact. Source-identifying portions were limited to lines containing hyperlinks, which were removed before blinded review. Both models operated on the same ChatGPT engine, and no stylistic modifications were made, minimizing the likelihood of recognizable differences. However, we did not formally assess whether raters could differentiate between the two models, which represents a minor limitation. Beyond this, the grounding of the model solely in IUGA patient information leaflets may limit generalizability across diverse cultural and linguistic contexts. Although all reviewers were board-certified urogynecologists, ensuring domain expertise, this may have introduced potential bias toward professional consensus. Also, the study did not assess patient perspectives, such as usability, readability, or trust, which are essential for understanding the real-world impact of the model and will be included in planned follow-up studies. Finally, large language models evolve rapidly, and our results represent a snapshot in time that may not reflect the performance of future model iterations. Nevertheless, this study contributes to a growing body of literature supporting the integration of retrieval-augmented AI in health communication.

Future research should assess how patients interpret and respond to AI-generated health content. Evaluating retrieval-augmented models with diverse patient populations, including those with limited health literacy or non-English language preferences, is essential to understanding real-world impact. Expanding the knowledge base to incorporate materials from additional professional societies may further enhance model performance. As a pilot study, our findings provide an early glimpse of the potential of retrieval-augmented AI to improve digital patient education in urogynecology.

In conclusion, integrating vetted patient-education materials into an AI framework significantly improved the quality of AI-generated responses in urogynecology. The retrieval-augmented ChatGPT model outperformed the standard model across all domains of the validated QAMAI assessment. These findings highlight the potential of retrieval-augmented AI models to improve the reliability of AI-generated medical information and underscore the importance of grounding AI tools in evidence-based resources to support informed, patient-centered care.

## Supplementary Information

Below is the link to the electronic supplementary material.

Supplementary file1 (DOCX 112 KB)

## Data Availability

Data will be shared upon request.
