# Supplementary material for: Development and Evaluation of an Augmented Artificial Intelligence Model for Urogynecology Queries
Source: Int Urogynecol J. 2025 Dec 9;37(5):1423–8. doi: 10.1007/s00192-025-06446-x (PMC13226325; doi:10.1007/s00192-025-06446-x)
Supplement: Supplementary file 1 — Supplementary file1 (DOCX 112 KB) [file 192_2025_6446_MOESM1_ESM.docx]

**Supplementary File**

1. Briefly tell me about the most important risks of bladder botox injections.

Option 1:

Bladder Botox injections are generally considered safe, but there are some important risks to be aware of:

1. **Urinary retention**: In some cases, Botox can cause difficulty in emptying the bladder, leading to urinary retention, which may require catheterization.
2. **Urinary tract infections (UTIs)**: Botox can increase the risk of UTIs, especially if there are issues with bladder emptying.
3. **Increased urgency or incontinence**: While Botox is meant to reduce bladder overactivity, it can sometimes cause side effects like increased urgency or incontinence.
4. **Pain or discomfort**: Some patients experience pain or discomfort at the injection site, including possible pelvic pain or bladder spasms.
5. **Allergic reaction**: Although rare, there's a small risk of allergic reactions to the Botox.
6. **Weakness in bladder muscles**: Overuse of Botox may weaken the bladder muscles over time, leading to further complications in bladder function.

Always consult with a healthcare provider to assess whether the benefits outweigh the risks for your specific condition.

Option 2:

Bladder Botox injections are a common treatment for overactive bladder or urinary incontinence when other options haven't worked. While generally safe and effective, there are some risks to consider:

1. **Temporary urinary retention** – Some patients may have difficulty emptying their bladder completely after the procedure. In some cases, intermittent self-catheterization may be needed until normal function returns.
2. **Urinary tract infection (UTI)** – There is a higher risk of developing a UTI after the injection.
3. **Hematuria (blood in the urine)** – A small amount of blood in the urine may occur temporarily after the procedure.
4. **General risks of Botox** – Rarely, Botox can spread beyond the bladder, potentially causing muscle weakness in other parts of the body.

It’s essential to discuss these risks with your healthcare provider to understand how they may apply to your situation. For more detailed information, you can refer to the IUGA Fact Sheet on [**Botulinum Toxin**](https://www.yourpelvicfloor.org/media/Botulinum_Toxin_English_Updated-RV3.pdf).

**Please select a rating in each category for response option 2.**

|  | Strongly Disagree | Disagree | Neutral | Agree | Strongly Agree |
| --- | --- | --- | --- | --- | --- |
| Provision of sources and references |  |  |  |  |  |

**Please select a rating in each category for response option 1.**

|  | Strongly Disagree | Disagree | Neutral | Agree | Strongly Agree |
| --- | --- | --- | --- | --- | --- |
| Provision of sources and references |  |  |  |  |  |

1. Briefly review risks of using vaginal estrogen.

Option 1:

Vaginal estrogen therapy is generally considered safe and effective for managing symptoms of genitourinary syndrome of menopause (GSM), such as vaginal dryness, irritation, painful intercourse, and urinary symptoms. Since the estrogen is applied locally, the amount absorbed into the bloodstream is minimal, which reduces the risk of systemic side effects.

However, potential risks include:

- **Local irritation:** Some women may experience mild vaginal irritation, discharge, or itching, particularly when starting treatment.
- **Bleeding:** Rarely, spotting or light bleeding may occur, especially in the early stages of use.
- **Infection:** Vaginal estrogen might slightly increase the risk of yeast infections in some women.
- **Precautions in certain conditions:** Women with a history of hormone-sensitive cancers (e.g., breast cancer) or blood clots should consult their healthcare provider before use.

Overall, the risks of vaginal estrogen are low, and most women tolerate it well. Always discuss your options with your healthcare provider to ensure it's the right treatment for you.

For more detailed information, refer to the **IUGA Fact Sheet on Low-Dose Vaginal Estrogen Therapy**: [Low-Dose Vaginal Estrogen Therapy](https://www.yourpelvicfloor.org/media/Low-Dose_Vaginal_Estrogen_Therapy.pdf).

Option 2:

Vaginal estrogen is commonly used to treat symptoms of vaginal atrophy (dryness, irritation) due to menopause or other estrogen deficiency conditions. While it is generally considered safe, it does carry some risks, including:

1. **Local side effects**: These may include vaginal irritation, discharge, or a mild burning sensation.
2. **Systemic absorption**: Though vaginal estrogen is thought to have minimal systemic absorption, it can still cause some systemic effects, particularly if used in high doses or over long periods. These effects may include:
   - **Breast tenderness** or enlargement
   - **Increased risk of blood clots** (though much lower than with oral estrogen)
   - **Potential impact on cardiovascular health** or endometrial tissue (though the local use significantly reduces these risks compared to systemic estrogen)
3. **Cancer risks**: There may be a slight increase in the risk of endometrial cancer, particularly if estrogen is used without progesterone in women with a uterus, though this is rare with low-dose vaginal estrogen.
4. **Infection risk**: Long-term use might increase the risk of urinary tract infections or yeast infections in some women.
5. **Pre-existing conditions**: Caution is advised in women with a history of breast cancer, blood clots, or liver disease, as vaginal estrogen may exacerbate these conditions.

Overall, vaginal estrogen is considered a safe and effective treatment with minimal systemic side effects, especially when used at low doses and for short durations. Regular follow-up with a healthcare provider is recommended to monitor any potential risks.

Using the following table, please rate question 1 – option 2.

|  | Strongly Disagree | Disagree | Neutral | Agree | Strongly Agree |
| --- | --- | --- | --- | --- | --- |
| Clarity |  |  |  |  |  |
| Relevance |  |  |  |  |  |
| Completeness |  |  |  |  |  |
| Usefulness |  |  |  |  |  |

Using the following table, please rate question 1 – option 1.

|  | Strongly Disagree | Disagree | Neutral | Agree | Strongly Agree |
| --- | --- | --- | --- | --- | --- |
| Clarity |  |  |  |  |  |
| Relevance |  |  |  |  |  |
| Completeness |  |  |  |  |  |
| Usefulness |  |  |  |  |  |

**Please select a rating in each category for response option 1.**

|  | Strongly Disagree | Disagree | Neutral | Agree | Strongly Agree |
| --- | --- | --- | --- | --- | --- |
| Provision of sources and references |  |  |  |  |  |

**Please select a rating in each category for response option 2.**

|  | Strongly Disagree | Disagree | Neutral | Agree | Strongly Agree |
| --- | --- | --- | --- | --- | --- |
| Provision of sources and references |  |  |  |  |  |

1. Briefly review maintenance for a pessary.

Option 1:

Proper maintenance of a pessary is essential to ensure its effectiveness, hygiene, and longevity. Here’s a brief overview:

1. **Cleaning**: A pessary should be cleaned regularly, typically once a week, using mild soap and water. Some women may be advised to use a specific disinfectant recommended by their healthcare provider. Avoid harsh chemicals or scented products that can irritate the vaginal tissues.
2. **Removal and Insertion**: Most women can remove and reinsert their pessary themselves, but they should be instructed on proper technique by a healthcare provider. Regular removal (usually once a week) helps prevent vaginal irritation, discharge, or infection.
3. **Inspection**: Periodic checks for signs of wear, damage, or irritation are important. If the pessary shows signs of cracking or other damage, it should be replaced.
4. **Follow-up visits**: Routine follow-up with a healthcare provider (typically every 3–6 months) is recommended to ensure the pessary is still fitting well and to monitor for any complications, such as vaginal irritation or prolapse recurrence.
5. **Hydration and lubrication**: If vaginal dryness is an issue, using a water-based lubricant when inserting the pessary can help avoid irritation.

By following these maintenance guidelines, women can help ensure their pessary remains effective and comfortable.

Option 2:

Proper maintenance of a vaginal pessary is essential to ensure it functions well, prevents complications, and maintains vaginal health.

**Key Steps for Pessary Maintenance:**

1. **Regular Cleaning:**
   - Depending on the type of pessary, it may need to be removed weekly or every few weeks for cleaning.
   - Wash the pessary with mild soap and water, rinse it thoroughly, and reinsert it as directed by your healthcare provider.
2. **Regular Follow-Ups:**
   - Visit your healthcare provider regularly (usually every 3–6 months) to check the fit and ensure there are no signs of irritation, infection, or pressure sores in the vaginal tissue.
3. **Use of Vaginal Estrogen (if recommended):**
   - Some women may benefit from low-dose vaginal estrogen to prevent vaginal dryness or thinning caused by pessary use, especially postmenopausal women.
4. **Watch for Symptoms:**
   - Contact your healthcare provider if you notice unusual discharge, odor, pain, or bleeding, as these may indicate infection or irritation.

Proper care ensures the pessary remains effective and comfortable for long-term use.

For more detailed guidance, refer to the **IUGA Fact Sheet on Vaginal Pessary for Pelvic Organ Prolapse**: [Vaginal Pessary for Pelvic Organ Prolapse](https://www.yourpelvicfloor.org/media/Vaginal_Pessary_for_Pelvic_Organ_Prolapse_RV2.pdf).

**Please select a rating in each category for response option 1.**

|  | Strongly Disagree | Disagree | Neutral | Agree | Strongly Agree |
| --- | --- | --- | --- | --- | --- |
| Provision of sources and references |  |  |  |  |  |

**Please select a rating in each category for response option 2.**

|  | Strongly Disagree | Disagree | Neutral | Agree | Strongly Agree |
| --- | --- | --- | --- | --- | --- |
| Provision of sources and references |  |  |  |  |  |

1. Briefly explain a sacrospinous ligament suspension versus a uterosacral ligament suspension.

Option 1:

Both **sacrospinous ligament suspension** and **uterosacral ligament suspension** are surgical procedures used to treat **pelvic organ prolapse** by providing support to the vaginal vault (top of the vagina) or uterus. Here's a brief comparison:

- **Sacrospinous Ligament Suspension**
- In this procedure, the top of the vagina is attached to the **sacrospinous ligament**, which is located near the tailbone (sacrum).
- It is typically done through the vagina (vaginal approach).
- This procedure is often used when the uterus has been removed (after a hysterectomy) or to support the vaginal apex.
- It provides excellent support but may slightly alter the vaginal axis (angle).
- For more details, see the [Sacrospinous Fixation Fact Sheet](https://www.yourpelvicfloor.org/media/Sacrospinous_Fixation_English_RV3-new.pdf).
- **Uterosacral Ligament Suspension**
- In this procedure, the vaginal apex or uterus is suspended to the **uterosacral ligaments**, which are located at the back of the pelvis and support the uterus naturally.
- It can be done through the vagina or the abdomen (laparoscopic or open surgery).
- This technique provides more "natural" anatomical support and maintains the vaginal axis better.
- For more details, see the [Uterosacral Ligament Suspension Fact Sheet](https://www.yourpelvicfloor.org/media/Uterosacral_Ligament_Suspension_RV3.pdf).

Both procedures are effective, and the choice depends on individual anatomy, the severity of the prolapse, and the surgeon's preference. Always discuss with your healthcare provider to determine the best option for you.

Option 2:

**Sacrospinous ligament suspension (SSLS)** and **uterosacral ligament suspension (USLS)** are surgical procedures used to treat pelvic organ prolapse, particularly for vaginal vault prolapse, by stabilizing the vaginal apex.

- **Sacrospinous ligament suspension (SSLS):** The vaginal apex is attached to the sacrospinous ligament, a strong ligament near the ischial spine. This procedure is typically performed vaginally and is effective for supporting the vaginal apex in cases of prolapse.
- **Uterosacral ligament suspension (USLS):** The vaginal apex or cervix is attached to the uterosacral ligaments, which are located near the sacrum. This procedure is commonly used for uterine prolapse and can also be done vaginally. It provides support by connecting the apex to a more posterior, stable structure.

Both aim to restore pelvic organ support, but SSLS is often preferred in post-hysterectomy cases, while USLS is more common in patients with an intact uterus.

**Please select a rating in each category for response option 1.**

|  | Strongly Disagree | Disagree | Neutral | Agree | Strongly Agree |
| --- | --- | --- | --- | --- | --- |
| Provision of sources and references |  |  |  |  |  |

**Please select a rating in each category for response option 2.**

|  | Strongly Disagree | Disagree | Neutral | Agree | Strongly Agree |
| --- | --- | --- | --- | --- | --- |
| Provision of sources and references |  |  |  |  |  |

1. Briefly review the most serious risk of a mid-urethral sling procedure.

Option 2:

The most serious risks of a **mid-urethral sling procedure** for stress urinary incontinence are rare but may include:

1. **Bladder or urethral injury** during surgery, which could require additional procedures to repair.
2. **Mesh complications**, such as mesh erosion (where the sling moves into surrounding tissues, like the vagina or urethra), infection, or pain.
3. **Difficulty urinating**, which might lead to urinary retention or the need for catheterization.
4. **Chronic pelvic pain** or discomfort in the vaginal area.
5. **Nerve or blood vessel damage**, which can occur during the placement of the sling.

It is essential to weigh these risks against the benefits of improved continence with your healthcare provider. Most complications can be managed effectively if they occur.

For more detailed information, you can consult the **IUGA Fact Sheet on Mid-Urethral Sling Procedures** [here](https://www.yourpelvicfloor.org/media/mid-urethral-slings-RV4.pdf).

Option 1:

The most serious risk of a mid-urethral sling procedure, typically used to treat stress urinary incontinence, is **bladder injury**. This can occur due to inadvertent placement of the sling, which may perforate the bladder or other nearby structures. Other notable risks include **infection**, **bleeding**, **chronic pain**, and **voiding dysfunction** (such as difficulty emptying the bladder). In some cases, the sling may also cause **mesh erosion** or **failure**, requiring additional surgery. While these risks are rare, they are important to consider when evaluating treatment options.

**Please select a rating in each category for response option 1.**

|  | Strongly Disagree | Disagree | Neutral | Agree | Strongly Agree |
| --- | --- | --- | --- | --- | --- |
| Provision of sources and references |  |  |  |  |  |

**Please select a rating in each category for response option 2.**

|  | Strongly Disagree | Disagree | Neutral | Agree | Strongly Agree |
| --- | --- | --- | --- | --- | --- |
| Provision of sources and references |  |  |  |  |  |

1. Briefly explain urodynamic testing.

Option 1:

Urodynamic testing is a series of tests used to evaluate how well the bladder, urethra, and sphincters are functioning. It helps diagnose urinary problems like incontinence, frequent urination, or difficulty urinating. The tests measure factors like bladder pressure, urine flow rate, and how well the bladder stores and empties urine. Common types of urodynamic tests include:

1. **Cystometry**: Measures bladder pressure and capacity.
2. **Uroflowmetry**: Measures the rate of urine flow.
3. **Pressure Flow Study**: Assesses bladder pressure during urination.
4. **Electromyography (EMG)**: Checks the function of the muscles involved in urination.

These tests are usually done in a specialized clinic or hospital.

Option 2:

Urodynamic testing is a set of tests used to evaluate how well the bladder, urethra, and pelvic floor muscles store and release urine. It helps diagnose problems like urinary incontinence, overactive bladder, or difficulty emptying the bladder. The tests may include measuring urine flow, bladder pressure, and how much urine the bladder can hold.

During the procedure, small catheters may be inserted into the bladder and rectum to monitor pressure, and you might be asked to urinate while measurements are taken. The test is generally safe and helps healthcare providers create a tailored treatment plan.

For more details, you can refer to the IUGA fact sheet: [Urodynamics](https://www.yourpelvicfloor.org/media/Urodynamics_RV2.pdf).

**Please select a rating in each category for response option 1.**

|  | Strongly Disagree | Disagree | Neutral | Agree | Strongly Agree |
| --- | --- | --- | --- | --- | --- |
| Provision of sources and references |  |  |  |  |  |

**Please select a rating in each category for response option 2.**

|  | Strongly Disagree | Disagree | Neutral | Agree | Strongly Agree |
| --- | --- | --- | --- | --- | --- |
| Provision of sources and references |  |  |  |  |  |

1. Briefly review how sacral neuromodulation works.

Option 1:

Sacral neuromodulation (SNM) is a treatment that involves stimulating the sacral nerves, which control the bladder, bowel, and pelvic floor muscles. It uses a small, implanted device (similar to a pacemaker) that sends mild electrical pulses to these nerves to help regulate their function. This therapy can be effective for conditions such as overactive bladder, urinary retention, and fecal incontinence when other treatments have not worked.

The procedure has two phases: a test phase to evaluate if the therapy works for the patient, followed by a permanent implant if it is successful. The goal is to restore normal communication between the brain and the pelvic organs, reducing symptoms and improving quality of life.

For more details, you can refer to the IUGA fact sheet on **Sacral Neuromodulation** here: [Sacral Neuromodulation](https://www.yourpelvicfloor.org/media/Sacral_Neuromodulation_RV4.pdf).

Option 2:

Sacral neuromodulation (SNM) is a therapeutic technique that involves stimulating the sacral nerves, which are located at the base of the spine, to regulate bladder, bowel, or pelvic floor function. It works by using electrical pulses delivered through a small implantable device, which is typically placed under the skin in the lower back or upper buttocks. These electrical signals modulate the nerve activity in the pelvic region, improving communication between the brain and the organs involved in urinary and bowel control.

SNM is often used to treat conditions such as overactive bladder, urinary incontinence, fecal incontinence, and interstitial cystitis, especially when other treatments have failed. The therapy is adjustable and reversible, making it a flexible option for many patients.

**Please select a rating in each category for response option 1.**

|  | Strongly Disagree | Disagree | Neutral | Agree | Strongly Agree |
| --- | --- | --- | --- | --- | --- |
| Provision of sources and references |  |  |  |  |  |

**Please select a rating in each category for response option 2.**

|  | Strongly Disagree | Disagree | Neutral | Agree | Strongly Agree |
| --- | --- | --- | --- | --- | --- |
| Provision of sources and references |  |  |  |  |  |

1. Briefly describe the best treatment for recurrent UTIs.

Option 1:

The best treatment for recurrent urinary tract infections (UTIs) typically involves a combination of approaches:

1. **Antibiotics**:
   - **Short-course therapy**: A brief course of antibiotics for each UTI episode (e.g., 3-7 days).
   - **Prophylactic antibiotics**: For frequent recurrences, low-dose antibiotics taken daily or after intercourse may be prescribed for several months.
2. **Self-start antibiotics**: Some patients may be given a prescription for antibiotics to start on their own when symptoms appear.
3. **Non-antibiotic options**:
   - **Urinary alkalinizers** or **cranberry products** (though evidence is mixed on their effectiveness).
   - **Probiotics**: Particularly *Lactobacillus* strains, which may help restore healthy vaginal flora.
4. **Lifestyle and hygiene changes**: Proper hydration, wiping front to back, urinating after intercourse, and avoiding irritants like scented products.

For the best management, it’s important to work with a healthcare provider to tailor treatment to the individual's needs.

Option 2:

The best treatment for recurrent urinary tract infections (UTIs) depends on the individual’s specific situation and the underlying cause of the recurrences. Common strategies include:

1. **Antibiotic Therapy**: Low-dose, long-term antibiotics or post-intercourse prophylaxis may be prescribed to prevent infections.
2. **Lifestyle Changes**: Staying hydrated, urinating after intercourse, and avoiding irritants like harsh soaps can help reduce recurrence.
3. **Vaginal Estrogen Therapy** (for postmenopausal women): Low-dose vaginal estrogen may improve vaginal health and reduce recurrent UTIs.
4. **Addressing Underlying Causes**: Treating conditions like incomplete bladder emptying or pelvic organ prolapse can prevent recurrences.

For more detailed guidance, see the IUGA fact sheet on [Urinary Tract Infections](https://www.yourpelvicfloor.org/media/Urinary_Tract_Infection_RV3.pdf). Always consult a healthcare provider for personalized advice.

**Please select a rating in each category for response option 1.**

|  | Strongly Disagree | Disagree | Neutral | Agree | Strongly Agree |
| --- | --- | --- | --- | --- | --- |
| Provision of sources and references |  |  |  |  |  |

**Please select a rating in each category for response option 2.**

|  | Strongly Disagree | Disagree | Neutral | Agree | Strongly Agree |
| --- | --- | --- | --- | --- | --- |
| Provision of sources and references |  |  |  |  |  |

1. Briefly review treatment options for vaginal prolapse.

Option 1:

Vaginal prolapse, also known as pelvic organ prolapse, can be managed with several treatment options depending on the severity of symptoms, your personal preferences, and your overall health. The main approaches include:

1. **Non-surgical options**:
   - **Pelvic floor exercises**: Strengthening the pelvic floor muscles can help reduce symptoms and prevent worsening of the prolapse. These are often guided by a physical therapist. [Pelvic Floor Exercises Fact Sheet](https://www.yourpelvicfloor.org/media/Pelvic_Floor_Exercises_RV2-1.pdf)
   - **Vaginal pessary**: A device inserted into the vagina to provide support to the prolapsed organs. It is non-invasive and can be a good option if surgery is not desired or recommended. [Vaginal Pessary for Pelvic Organ Prolapse Fact Sheet](https://www.yourpelvicfloor.org/media/Vaginal_Pessary_for_Pelvic_Organ_Prolapse_RV2.pdf)
2. **Surgical options**:
   - **Anterior or posterior vaginal wall repair**: Surgery to repair the vaginal walls and provide better support to the prolapsed organs. [Anterior Vaginal Repair Fact Sheet](https://www.yourpelvicfloor.org/media/anterior-vaginal-wall-repair-RV3.pdf)
     [Posterior Vaginal Wall Repair Fact Sheet](https://www.yourpelvicfloor.org/media/posterior-vaginal-wall-and-perineal-body-repair-RV2-1.pdf)
   - **Uterine-preserving surgeries**: If you wish to retain your uterus, procedures such as sacrohysteropexy or uterosacral ligament suspension can provide support while maintaining uterine integrity. [Uterine Preserving Surgery Fact Sheet](https://www.yourpelvicfloor.org/media/uterine-preservation-RV1.pdf)
   - **Vaginal hysterectomy**: If the uterus is prolapsed, it may be removed, often with additional repairs to strengthen support structures. [Vaginal Hysterectomy for Prolapse Fact Sheet](https://www.yourpelvicfloor.org/media/vaginal-hysterectomy-for-pelvic-organ-prolapse-RV2-1.pdf)
   - **Sacrocolpopexy**: A procedure where mesh is used to support the vagina and attach it to the sacrum for long-term support. [Sacrocolpopexy Fact Sheet](https://www.yourpelvicfloor.org/media/sacrocolpopexy-RV3.pdf)

Each option has its own benefits and risks. It’s essential to discuss these with your healthcare provider to decide on the best approach for you. You can also read the IUGA fact sheet on [**Pelvic Organ Prolapse**](https://www.yourpelvicfloor.org/media/Pelvic_Organ_Prolapse_RV3.pdf) for more detailed information.

Option 2:

Vaginal prolapse, also known as pelvic organ prolapse, occurs when the pelvic organs (such as the bladder, uterus, or rectum) descend into the vaginal canal due to weakened pelvic floor muscles. Treatment options depend on the severity of the prolapse, symptoms, and individual health factors. Here are the main options:

1. **Conservative Treatments**:
   - **Pelvic floor exercises (Kegel exercises)**: Strengthening the pelvic muscles can help support the prolapsed organs.
   - **Pessaries**: These are devices inserted into the vagina to support the prolapsed organs. They're non-surgical and can be effective for managing symptoms.
2. **Medical Treatments**:
   - **Estrogen therapy**: In postmenopausal women, vaginal estrogen may help improve the strength and elasticity of vaginal tissues, improving support for the pelvic organs.
3. **Surgical Treatments**:
   - **Vaginal surgery**: Procedures such as a vaginal hysterectomy (removal of the uterus) or anterior/posterior vaginal repair (to repair the bladder or rectum prolapse) can be done to reposition the prolapsed organs.
   - **Laparoscopic surgery**: Minimally invasive techniques can be used for prolapse repair with shorter recovery times.
   - **Sacral colpopexy**: A surgery that uses mesh to support the vagina and pelvic organs, often used for more severe prolapse.

The treatment decision depends on the extent of the prolapse, symptoms, and your overall health and preferences. A healthcare provider, such as a gynecologist or urologist, can help determine the best approach for your individual case.

**Please select a rating in each category for response option 1.**

|  | Strongly Disagree | Disagree | Neutral | Agree | Strongly Agree |
| --- | --- | --- | --- | --- | --- |
| Provision of sources and references |  |  |  |  |  |

**Please select a rating in each category for response option 2.**

|  | Strongly Disagree | Disagree | Neutral | Agree | Strongly Agree |
| --- | --- | --- | --- | --- | --- |
| Provision of sources and references |  |  |  |  |  |

1. Briefly describe risks of overactive bladder medications.

Option 1:

Overactive bladder (OAB) medications, like antimuscarinics (e.g., oxybutynin, tolterodine) and beta-3 adrenergic agonists (e.g., mirabegron), can have several risks, including:

1. **Dry Mouth**: A common side effect of antimuscarinics due to reduced saliva production.
2. **Constipation**: Another side effect, particularly with antimuscarinics, as they can slow gastrointestinal motility.
3. **Blurred Vision**: Caused by the antimuscarinic effect on the eyes, impairing accommodation.
4. **Urinary Retention**: Beta-3 agonists like mirabegron can cause difficulty emptying the bladder in some individuals.
5. **Cognitive Effects**: Older adults are at higher risk for confusion, memory problems, or delirium with antimuscarinics.
6. **Elevated Blood Pressure**: Mirabegron can raise blood pressure in some individuals, requiring monitoring.
7. **Cardiovascular Risks**: Some medications may affect heart rate or cause arrhythmias, particularly in those with pre-existing heart conditions.

Always consult with a healthcare provider to evaluate risks and benefits.

Option 2:

Medications for overactive bladder (OAB), such as anticholinergics and beta-3 adrenergic agonists, can be effective but may come with some risks and side effects:

1. **Anticholinergic Medications**:
   - Common side effects include dry mouth, constipation, blurred vision, and dizziness.
   - They may also cause urinary retention (difficulty emptying the bladder).
   - In older adults, these medications can increase the risk of confusion, memory problems, or cognitive decline.
2. **Beta-3 Adrenergic Agonists (e.g., Mirabegron)**:
   - Possible side effects include increased blood pressure, headache, and urinary tract infections (UTIs).
   - They may not be suitable for patients with uncontrolled high blood pressure.

It's important to discuss with your healthcare provider whether these medications are appropriate for you, considering your health history and any potential interactions with other medications.

For more information about managing overactive bladder, you can refer to the IUGA fact sheet: [Overactive Bladder](https://www.yourpelvicfloor.org/media/Overactive_Bladder_RV2.pdf).

**Please select a rating in each category for response option 1.**

|  | Strongly Disagree | Disagree | Neutral | Agree | Strongly Agree |
| --- | --- | --- | --- | --- | --- |
| Provision of sources and references |  |  |  |  |  |

**Please select a rating in each category for response option 2.**

|  | Strongly Disagree | Disagree | Neutral | Agree | Strongly Agree |
| --- | --- | --- | --- | --- | --- |
| Provision of sources and references |  |  |  |  |  |
